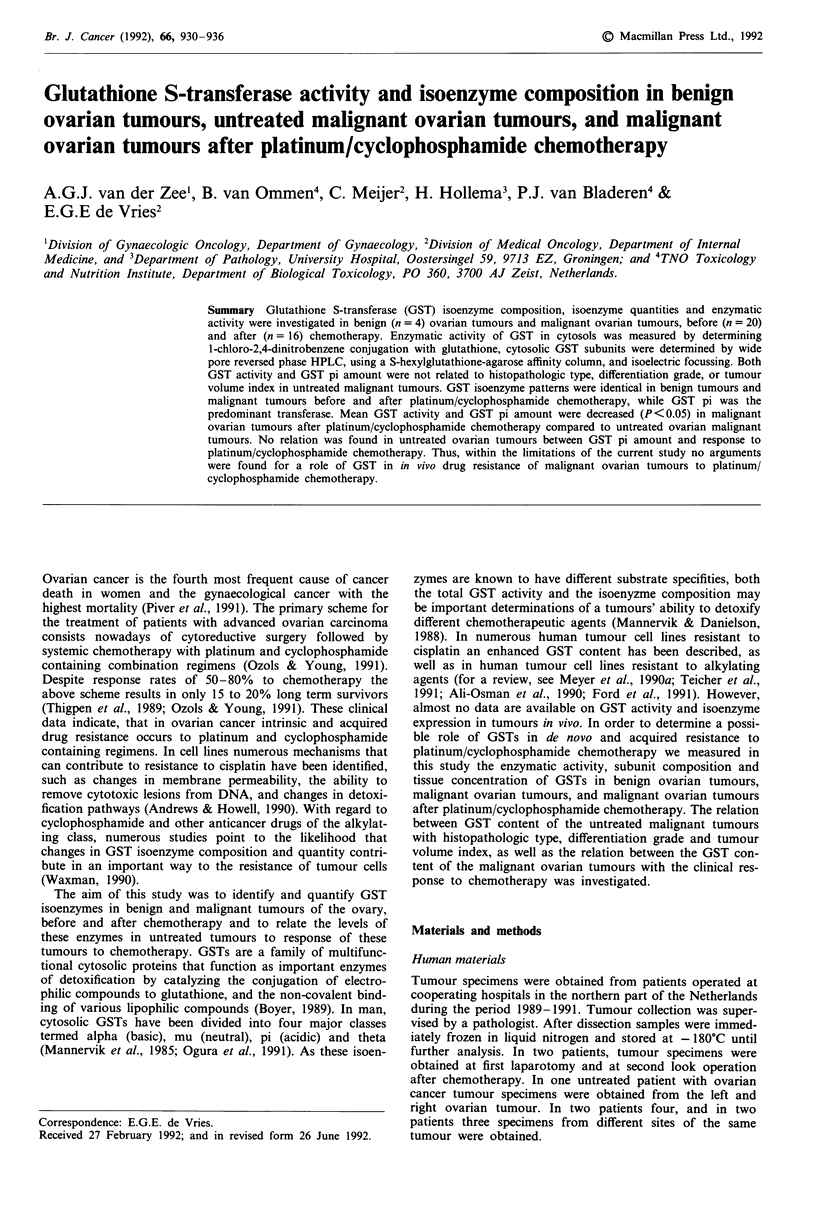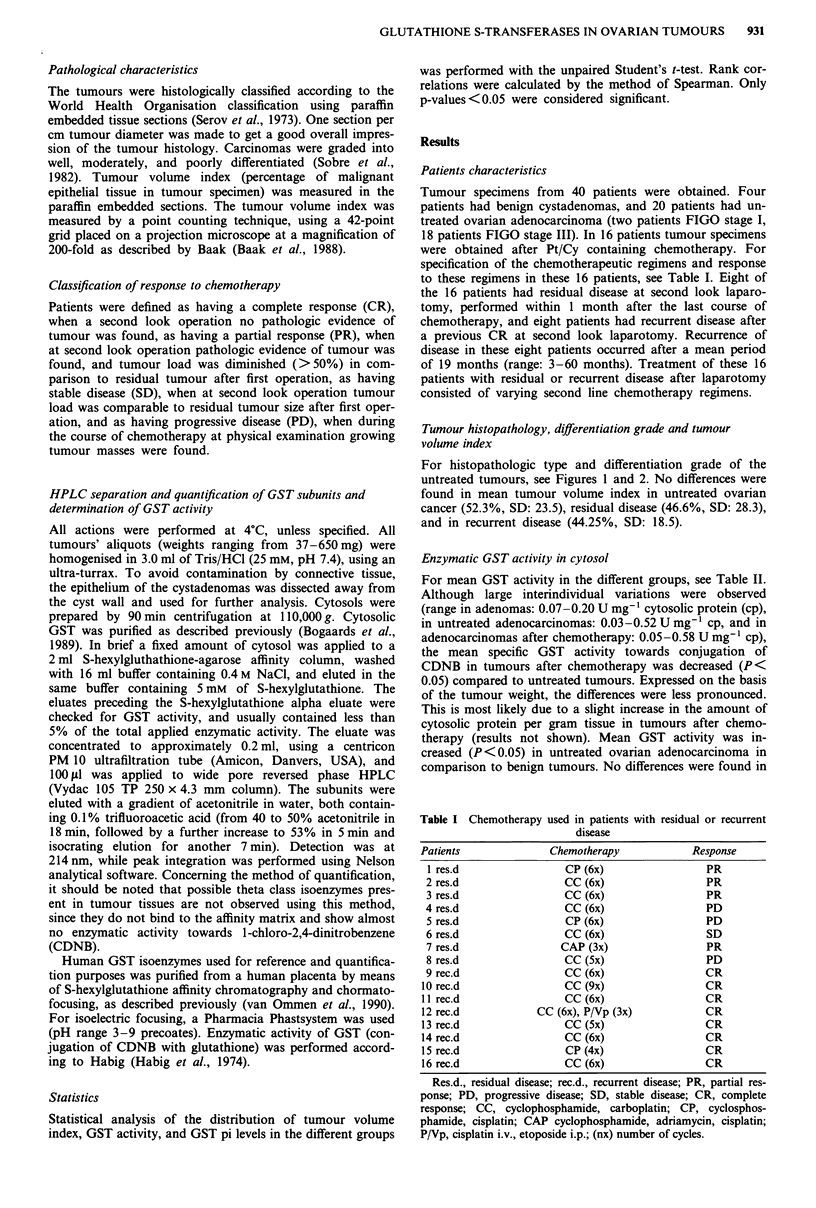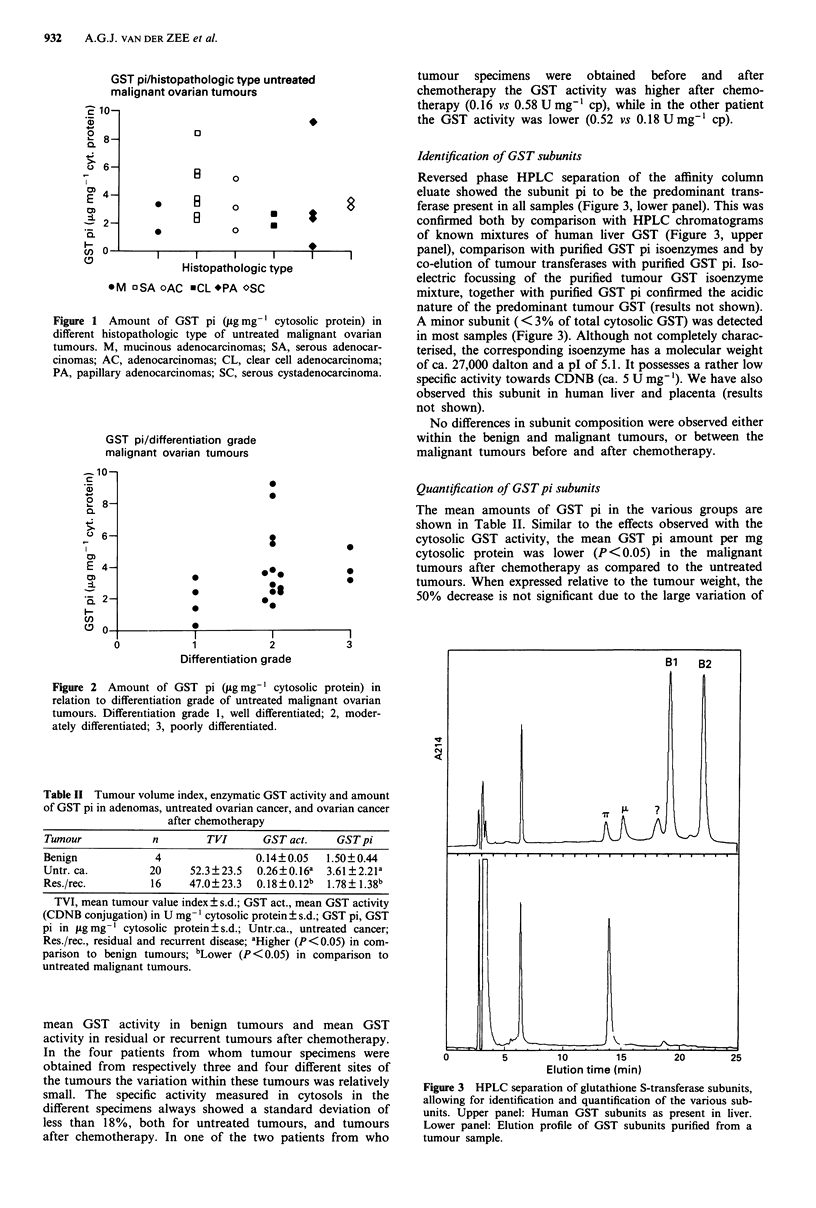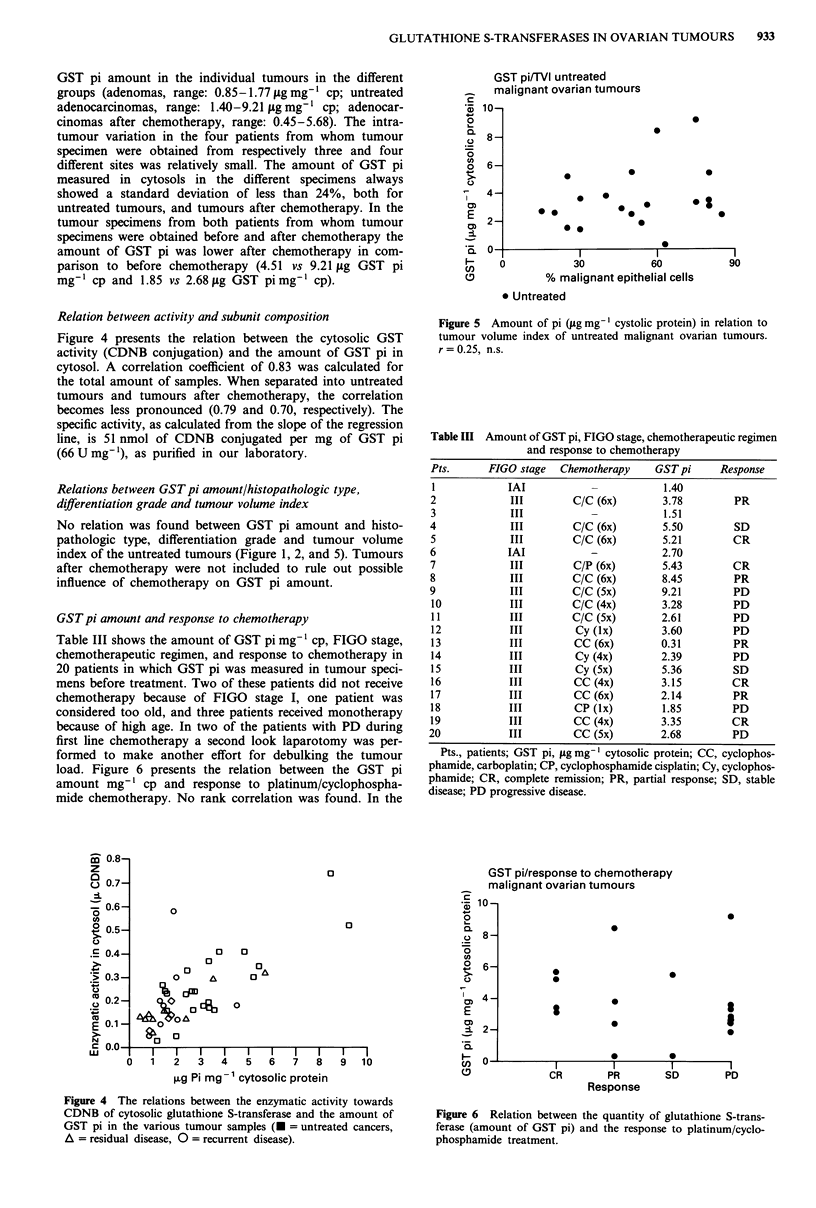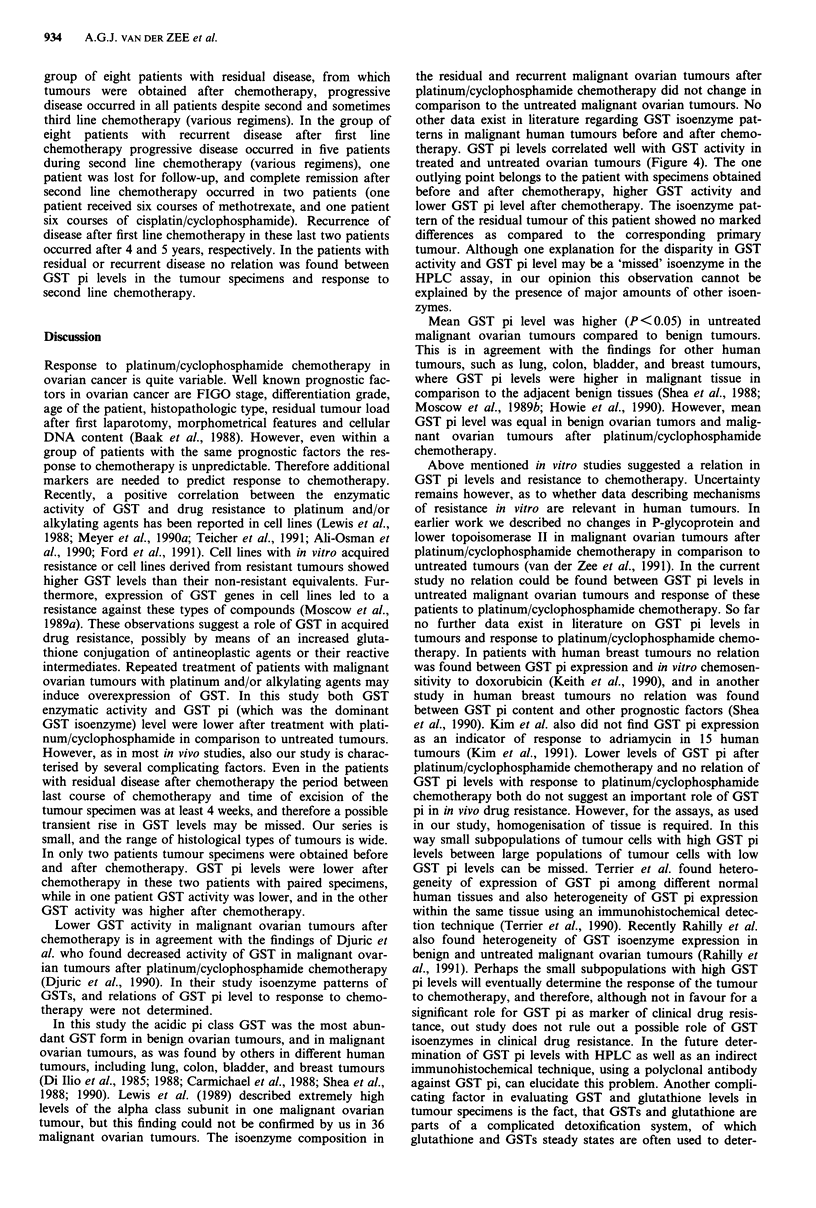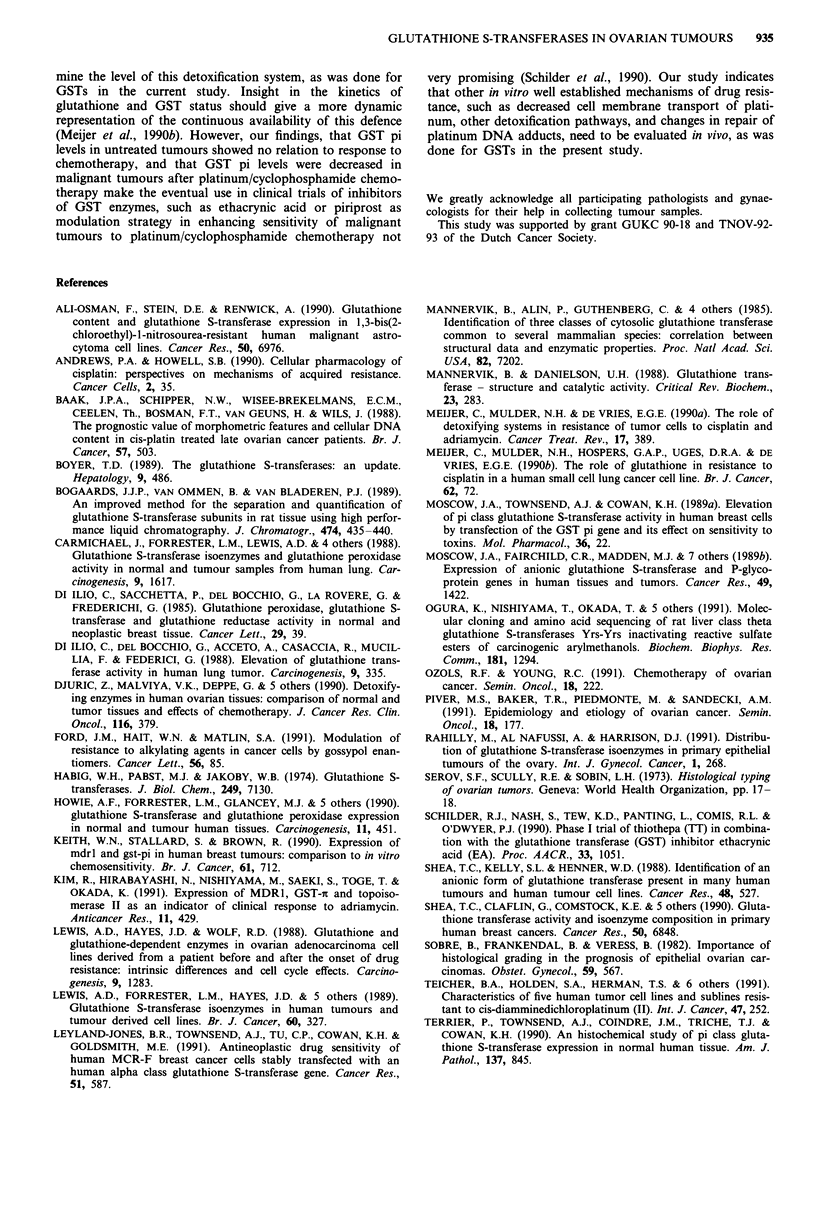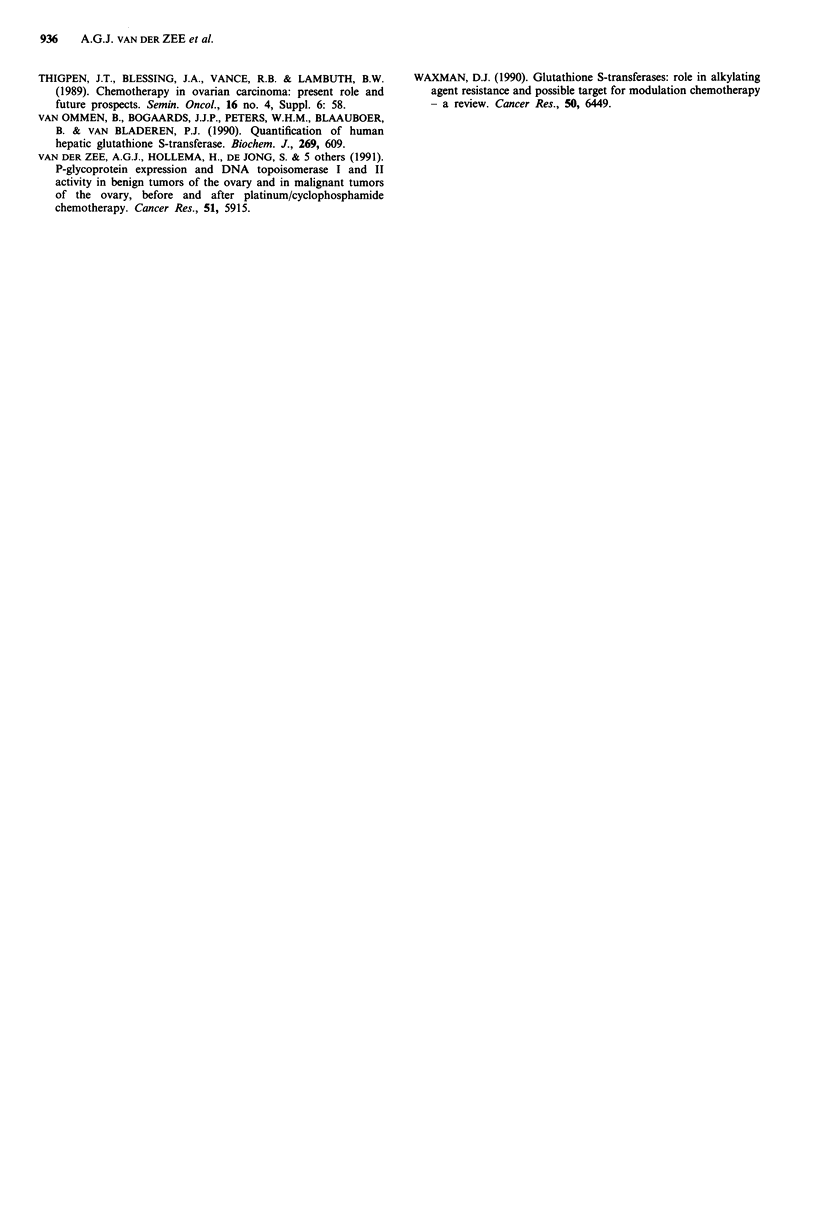# Glutathione S-transferase activity and isoenzyme composition in benign ovarian tumours, untreated malignant ovarian tumours, and malignant ovarian tumours after platinum/cyclophosphamide chemotherapy.

**DOI:** 10.1038/bjc.1992.388

**Published:** 1992-11

**Authors:** A. G. van der Zee, B. van Ommen, C. Meijer, H. Hollema, P. J. van Bladeren, E. G. de Vries

**Affiliations:** Department of Gynaecology, University Hospital, Groningen, Netherlands.

## Abstract

Glutathione S-transferase (GST) isoenzyme composition, isoenzyme quantities and enzymatic activity were investigated in benign (n = 4) ovarian tumours and malignant ovarian tumours, before (n = 20) and after (n = 16) chemotherapy. Enzymatic activity of GST in cytosols was measured by determining 1-chloro-2,4-dinitrobenzene conjugation with glutathione, cytosolic GST subunits were determined by wide pore reversed phase HPLC, using a S-hexylglutathione-agarose affinity column, and isoelectric focussing. Both GST activity and GST pi amount were not related to histopathologic type, differentiation grade, or tumour volume index in untreated malignant tumours. GST isoenzyme patterns were identical in benign tumours and malignant tumours before and after platinum/cyclophosphamide chemotherapy, while GST pi was the predominant transferase. Mean GST activity and GST pi amount were decreased (P < 0.05) in malignant ovarian tumours after platinum/cyclophosphamide chemotherapy compared to untreated ovarian malignant tumours. No relation was found in untreated ovarian tumours between GST pi amount and response to platinum/cyclophosphamide chemotherapy. Thus, within the limitations of the current study no arguments were found for a role of GST in in vivo drug resistance of malignant ovarian tumours to platinum/cyclophosphamide chemotherapy.


 © Macmillan Press Ltd., 1992

### Glutathione S-transferase activity and isoenzyme composition in benign ovarian tumours, untreated malignant ovarian tumours, and malignant ovarian tumours after platinum/cyclophosphamide chemotherapy

A.G.J. van der Zee[1], B. van Ommen[4], C. Meijer[2], H. Hollema[3], P.J. van Bladeren[4] & E.G.E de Vries[2]

[1]*Division of Gynaecologic Oncology, Department of Gynaecology,* [2]*Division of Medical Oncology, Department of Internal Medicine, and* [3]*Department of Pathology, University Hospital, Oostersingel 59, 9713 EZ, Groningen; and* [4]*TNO Toxicology and Nutrition Institute, Department of Biological Toxicology, PO 360, 3700 AJ Zeist, Netherlands.*

**Summary** Glutathione S-transferase (GST) isoenzyme composition, isoenzyme quantities and enzymatic activity were investigated in benign ($n = 4$) ovarian tumours and malignant ovarian tumours, before ($n = 20$) and after ($n = 16$) chemotherapy. Enzymatic activity of GST in cytosols was measured by determining 1-chloro-2,4-dinitrobenzene conjugation with glutathione, cytosolic GST subunits were determined by wide pore reversed phase HPLC, using a S-hexylglutathione-agarose affinity column, and isoelectric focussing. Both GST activity and GST pi amount were not related to histopathologic type, differentiation grade, or tumour volume index in untreated malignant tumours. GST isoenzyme patterns were identical in benign tumours and malignant tumours before and after platinum/cyclophosphamide chemotherapy, while GST pi was the predominant transferase. Mean GST activity and GST pi amount were decreased ($P < 0.05$) in malignant ovarian tumours after platinum/cyclophosphamide chemotherapy compared to untreated ovarian malignant tumours. No relation was found in untreated ovarian tumours between GST pi amount and response to platinum/cyclophosphamide chemotherapy. Thus, within the limitations of the current study no arguments were found for a role of GST in *in vivo* drug resistance of malignant ovarian tumours to platinum/cyclophosphamide chemotherapy.

Ovarian cancer is the fourth most frequent cause of cancer death in women and the gynaecological cancer with the highest mortality (Piver *et al.*, 1991). The primary scheme for the treatment of patients with advanced ovarian carcinoma consists nowadays of cytoreductive surgery followed by systemic chemotherapy with platinum and cyclophosphamide containing combination regimens (Ozols & Young, 1991). Despite response rates of 50-80% to chemotherapy the above scheme results in only 15 to 20% long term survivors (Thigpen *et al.*, 1989; Ozols & Young, 1991). These clinical data indicate, that in ovarian cancer intrinsic and acquired drug resistance occurs to platinum and cyclophosphamide containing regimens. In cell lines numerous mechanisms that can contribute to resistance to cisplatin have been identified, such as changes in membrane permeability, the ability to remove cytotoxic lesions from DNA, and changes in detoxification pathways (Andrews & Howell, 1990). With regard to cyclophosphamide and other anticancer drugs of the alkylating class, numerous studies point to the likelihood that changes in GST isoenzyme composition and quantity contribute in an important way to the resistance of tumour cells (Waxman, 1990).

The aim of this study was to identify and quantify GST isoenzymes in benign and malignant tumours of the ovary, before and after chemotherapy and to relate the levels of these enzymes in untreated tumours to response of these tumours to chemotherapy. GSTs are a family of multifunctional cytosolic proteins that function as important enzymes of detoxification by catalyzing the conjugation of electrophilic compounds to glutathione, and the non-covalent binding of various lipophilic compounds (Boyer, 1989). In man, cytosolic GSTs have been divided into four major classes termed alpha (basic), mu (neutral), pi (acidic) and theta (Mannervik *et al.*, 1985; Ogura *et al.*, 1991). As these isoenzymes are known to have different substrate specificities, both the total GST activity and the isoenyzme composition may be important determinations of a tumours' ability to detoxify different chemotherapeutic agents (Mannervik & Danielson, 1988). In numerous human tumour cell lines resistant to cisplatin an enhanced GST content has been described, as well as in human tumour cell lines resistant to alkylating agents (for a review, see Meyer *et al.*, 1990*a*; Teicher *et al.*, 1991; Ali-Osman *et al.*, 1990; Ford *et al.*, 1991). However, almost no data are available on GST activity and isoenzyme expression in tumours *in vivo*. In order to determine a possible role of GSTs in *de novo* and acquired resistance to platinum/cyclophosphamide chemotherapy we measured in this study the enzymatic activity, subunit composition and tissue concentration of GSTs in benign ovarian tumours, malignant ovarian tumours, and malignant ovarian tumours after platinum/cyclophosphamide chemotherapy. The relation between GST content of the untreated malignant tumours with histopathologic type, differentiation grade and tumour volume index, as well as the relation between the GST content of the malignant ovarian tumours with the clinical response to chemotherapy was investigated.

### Materials and methods

### *Human materials*

Tumour specimens were obtained from patients operated at cooperating hospitals in the northern part of the Netherlands during the period 1989-1991. Tumour collection was supervised by a pathologist. After dissection samples were immediately frozen in liquid nitrogen and stored at −180°C until further analysis. In two patients, tumour specimens were obtained at first laparotomy and at second look operation after chemotherapy. In one untreated patient with ovarian cancer tumour specimens were obtained from the left and right ovarian tumour. In two patients four, and in two patients three specimens from different sites of the same tumour were obtained.

Correspondence: E.G.E. de Vries.
Received 27 February 1992; and in revised form 26 June 1992.

*Pathological characteristics*

The tumours were histologically classified according to the World Health Organisation classification using paraffin embedded tissue sections (Serov *et al.*, 1973). One section per cm tumour diameter was made to get a good overall impression of the tumour histology. Carcinomas were graded into well, moderately, and poorly differentiated (Sobre *et al.*, 1982). Tumour volume index (percentage of malignant epithelial tissue in tumour specimen) was measured in the paraffin embedded sections. The tumour volume index was measured by a point counting technique, using a 42-point grid placed on a projection microscope at a magnification of 200-fold as described by Baak (Baak *et al.*, 1988).

*Classification of response to chemotherapy*

Patients were defined as having a complete response (CR), when a second look operation no pathologic evidence of tumour was found, as having a partial response (PR), when at second look operation pathologic evidence of tumour was found, and tumour load was diminished (>50%) in comparison to residual tumour after first operation, as having stable disease (SD), when at second look operation tumour load was comparable to residual tumour size after first operation, and as having progressive disease (PD), when during the course of chemotherapy at physical examination growing tumour masses were found.

*HPLC separation and quantification of GST subunits and determination of GST activity*

All actions were performed at 4°C, unless specified. All tumours' aliquots (weights ranging from 37–650 mg) were homogenised in 3.0 ml of Tris/HCl (25 mM, pH 7.4), using an ultra-turrax. To avoid contamination by connective tissue, the epithelium of the cystadenomas was dissected away from the cyst wall and used for further analysis. Cytosols were prepared by 90 min centrifugation at 110,000 *g*. Cytosolic GST was purified as described previously (Bogaards *et al.*, 1989). In brief a fixed amount of cytosol was applied to a 2 ml S-hexylgluthathione-agarose affinity column, washed with 16 ml buffer containing 0.4 M NaCl, and eluted in the same buffer containing 5 mM of S-hexylglutathione. The eluates preceding the S-hexylglutathione alpha eluate were checked for GST activity, and usually contained less than 5% of the total applied enzymatic activity. The eluate was concentrated to approximately 0.2 ml, using a centricon PM 10 ultrafiltration tube (Amicon, Danvers, USA), and 100 µl was applied to wide pore reversed phase HPLC (Vydac 105 TP 250 × 4.3 mm column). The subunits were eluted with a gradient of acetonitrile in water, both containing 0.1% trifluoroacetic acid (from 40 to 50% acetonitrile in 18 min, followed by a further increase to 53% in 5 min and isocrating elution for another 7 min). Detection was at 214 nm, while peak integration was performed using Nelson analytical software. Concerning the method of quantification, it should be noted that possible theta class isoenzymes present in tumour tissues are not observed using this method, since they do not bind to the affinity matrix and show almost no enzymatic activity towards 1-chloro-2,4-dinitrobenzene (CDNB).

Human GST isoenzymes used for reference and quantification purposes was purified from a human placenta by means of S-hexylglutathione affinity chromatography and chormatofocusing, as described previously (van Ommen *et al.*, 1990). For isoelectric focusing, a Pharmacia Phastsystem was used (pH range 3–9 precoates). Enzymatic activity of GST (conjugation of CDNB with glutathione) was performed according to Habig (Habig *et al.*, 1974).

*Statistics*

Statistical analysis of the distribution of tumour volume index, GST activity, and GST pi levels in the different groups was performed with the unpaired Student's *t*-test. Rank correlations were calculated by the method of Spearman. Only p-values $<0.05$ were considered significant.

### Results

*Patients characteristics*

Tumour specimens from 40 patients were obtained. Four patients had benign cystadenomas, and 20 patients had untreated ovarian adenocarcinoma (two patients FIGO stage I, 18 patients FIGO stage III). In 16 patients tumour specimens were obtained after Pt/Cy containing chemotherapy. For specification of the chemotherapeutic regimens and response to these regimens in these 16 patients, see Table I. Eight of the 16 patients had residual disease at second look laparotomy, performed within 1 month after the last course of chemotherapy, and eight patients had recurrent disease after a previous CR at second look laparotomy. Recurrence of disease in these eight patients occurred after a mean period of 19 months (range: 3–60 months). Treatment of these 16 patients with residual or recurrent disease after laparotomy consisted of varying second line chemotherapy regimens.

*Tumour histopathology, differentiation grade and tumour volume index*

For histopathologic type and differentiation grade of the untreated tumours, see Figures 1 and 2. No differences were found in mean tumour volume index in untreated ovarian cancer (52.3%, SD: 23.5), residual disease (46.6%, SD: 28.3), and in recurrent disease (44.25%, SD: 18.5).

*Enzymatic GST activity in cytosol*

For mean GST activity in the different groups, see Table II. Although large interindividual variations were observed (range in adenomas: 0.07–0.20 U $mg^{-1}$ cytosolic protein (cp), in untreated adenocarcinomas: 0.03–0.52 U $mg^{-1}$ cp, and in adenocarcinomas after chemotherapy: 0.05–0.58 U $mg^{-1}$ cp), the mean specific GST activity towards conjugation of CDNB in tumours after chemotherapy was decreased ($P<0.05$) compared to untreated tumours. Expressed on the basis of the tumour weight, the differences were less pronounced. This is most likely due to a slight increase in the amount of cytosolic protein per gram tissue in tumours after chemotherapy (results not shown). Mean GST activity was increased ($P<0.05$) in untreated ovarian adenocarcinoma in comparison to benign tumours. No differences were found in

**Table I** Chemotherapy used in patients with residual or recurrent disease

| *Patients* | *Chemotherapy* | *Response* |
|---|---|---|
| 1 res.d | CP (6x) | PR |
| 2 res.d | CC (6x) | PR |
| 3 res.d | CC (6x) | PR |
| 4 res.d | CC (6x) | PD |
| 5 res.d | CP (6x) | PD |
| 6 res.d | CC (6x) | SD |
| 7 res.d | CAP (3x) | PR |
| 8 res.d | CC (5x) | PD |
| 9 rec.d | CC (6x) | CR |
| 10 rec.d | CC (9x) | CR |
| 11 rec.d | CC (6x) | CR |
| 12 rec.d | CC (6x), P/Vp (3x) | CR |
| 13 rec.d | CC (5x) | CR |
| 14 rec.d | CC (6x) | CR |
| 15 rec.d | CP (4x) | CR |
| 16 rec.d | CC (6x) | CR |

Res.d., residual disease; rec.d., recurrent disease; PR, partial response; PD, progressive disease; SD, stable disease; CR, complete response; CC, cyclophosphamide, carboplatin; CP, cyclosphosphamide, cisplatin; CAP cyclophosphamide, adriamycin, cisplatin; P/Vp, cisplatin i.v., etoposide i.p.; (nx) number of cycles.

**Figure 1** Amount of GST pi (μg mg$^{-1}$ cytosolic protein) in different histopathologic type of untreated malignant ovarian tumours. M, mucinous adenocarcinomas; SA, serous adenocarcinomas; AC, adenocarcinomas; CL, clear cell adenocarcinoma; PA, papillary adenocarcinomas; SC, serous cystadenocarcinoma.

**Figure 2** Amount of GST pi (μg mg$^{-1}$ cytosolic protein) in relation to differentiation grade of untreated malignant ovarian tumours. Differentiation grade 1, well differentiated; 2, moderately differentiated; 3, poorly differentiated.

**Table II** Tumour volume index, enzymatic GST activity and amount of GST pi in adenomas, untreated ovarian cancer, and ovarian cancer after chemotherapy

| *Tumour* | *n* | *TVI* | *GST act.* | *GST pi* |
|---|---|---|---|---|
| Benign | 4 | | 0.14±0.05 | 1.50±0.44 |
| Untr. ca. | 20 | 52.3±23.5 | 0.26±0.16[a] | 3.61±2.21[a] |
| Res./rec. | 16 | 47.0±23.3 | 0.18±0.12[b] | 1.78±1.38[b] |

TVI, mean tumour value index±s.d.; GST act., mean GST activity (CDNB conjugation) in U mg$^{-1}$ cytosolic protein±s.d.; GST pi, GST pi in μg mg$^{-1}$ cytosolic protein±s.d.; Untr.ca., untreated cancer; Res./rec., residual and recurrent disease; [a]Higher ($P<0.05$) in comparison to benign tumours; [b]Lower ($P<0.05$) in comparison to untreated malignant tumours.

mean GST activity in benign tumours and mean GST activity in residual or recurrent tumours after chemotherapy. In the four patients from whom tumour specimens were obtained from respectively three and four different sites of the tumours the variation within these tumours was relatively small. The specific activity measured in cytosols in the different specimens always showed a standard deviation of less than 18%, both for untreated tumours, and tumours after chemotherapy. In one of the two patients from who tumour specimens were obtained before and after chemotherapy the GST activity was higher after chemotherapy (0.16 *vs* 0.58 U mg$^{-1}$ cp), while in the other patient the GST activity was lower (0.52 *vs* 0.18 U mg$^{-1}$ cp).

*Identification of GST subunits*

Reversed phase HPLC separation of the affinity column eluate showed the subunit pi to be the predominant transferase present in all samples (Figure 3, lower panel). This was confirmed both by comparison with HPLC chromatograms of known mixtures of human liver GST (Figure 3, upper panel), comparison with purified GST pi isoenzymes and by co-elution of tumour transferases with purified GST pi. Isoelectric focussing of the purified tumour GST isoenzyme mixture, together with purified GST pi confirmed the acidic nature of the predominant tumour GST (results not shown). A minor subunit (<3% of total cytosolic GST) was detected in most samples (Figure 3). Although not completely characterised, the corresponding isoenzyme has a molecular weight of ca. 27,000 dalton and a pI of 5.1. It possesses a rather low specific activity towards CDNB (ca. 5 U mg$^{-1}$). We have also observed this subunit in human liver and placenta (results not shown).

No differences in subunit composition were observed either within the benign and malignant tumours, or between the malignant tumours before and after chemotherapy.

*Quantification of GST pi subunits*

The mean amounts of GST pi in the various groups are shown in Table II. Similar to the effects observed with the cytosolic GST activity, the mean GST pi amount per mg cytosolic protein was lower ($P<0.05$) in the malignant tumours after chemotherapy as compared to the untreated tumours. When expressed relative to the tumour weight, the 50% decrease is not significant due to the large variation of

**Figure 3** HPLC separation of glutathione S-transferase subunits, allowing for identification and quantification of the various subunits. Upper panel: Human GST subunits as present in liver. Lower panel: Elution profile of GST subunits purified from a tumour sample.

GST pi amount in the individual tumours in the different groups (adenomas, range: 0.85–1.77 μg mg$^{-1}$ cp; untreated adenocarcinomas, range: 1.40–9.21 μg mg$^{-1}$ cp; adenocarcinomas after chemotherapy, range: 0.45–5.68). The intratumour variation in the four patients from whom tumour specimen were obtained from respectively three and four different sites was relatively small. The amount of GST pi measured in cytosols in the different specimens always showed a standard deviation of less than 24%, both for untreated tumours, and tumours after chemotherapy. In the tumour specimens from both patients from whom tumour specimens were obtained before and after chemotherapy the amount of GST pi was lower after chemotherapy in comparison to before chemotherapy (4.51 *vs* 9.21 μg GST pi mg$^{-1}$ cp and 1.85 *vs* 2.68 μg GST pi mg$^{-1}$ cp).

### *Relation between activity and subunit composition*

Figure 4 presents the relation between the cytosolic GST activity (CDNB conjugation) and the amount of GST pi in cytosol. A correlation coefficient of 0.83 was calculated for the total amount of samples. When separated into untreated tumours and tumours after chemotherapy, the correlation becomes less pronounced (0.79 and 0.70, respectively). The specific activity, as calculated from the slope of the regression line, is 51 nmol of CDNB conjugated per mg of GST pi (66 U mg$^{-1}$), as purified in our laboratory.

### *Relations between GST pi amount/histopathologic type, differentiation grade and tumour volume index*

No relation was found between GST pi amount and histopathologic type, differentiation grade and tumour volume index of the untreated tumours (Figure 1, 2, and 5). Tumours after chemotherapy were not included to rule out possible influence of chemotherapy on GST pi amount.

### *GST pi amount and response to chemotherapy*

Table III shows the amount of GST pi mg$^{-1}$ cp, FIGO stage, chemotherapeutic regimen, and response to chemotherapy in 20 patients in which GST pi was measured in tumour specimens before treatment. Two of these patients did not receive chemotherapy because of FIGO stage I, one patient was considered too old, and three patients received monotherapy because of high age. In two of the patients with PD during first line chemotherapy a second look laparotomy was performed to make another effort for debulking the tumour load. Figure 6 presents the relation between the GST pi amount mg$^{-1}$ cp and response to platinum/cyclophosphamide chemotherapy. No rank correlation was found. In the

**Figure 5** Amount of pi (μg mg$^{-1}$ cystolic protein) in relation to tumour volume index of untreated malignant ovarian tumours. $r = 0.25$, n.s.

**Table III** Amount of GST pi, FIGO stage, chemotherapeutic regimen and response to chemotherapy

| *Pts.* | *FIGO stage* | *Chemotherapy* | *GST pi* | *Response* |
|---|---|---|---|---|
| 1 | IAI | – | 1.40 | |
| 2 | III | C/C (6x) | 3.78 | PR |
| 3 | III | – | 1.51 | |
| 4 | III | C/C (6x) | 5.50 | SD |
| 5 | III | C/C (6x) | 5.21 | CR |
| 6 | IAI | – | 2.70 | |
| 7 | III | C/P (6x) | 5.43 | CR |
| 8 | III | C/C (6x) | 8.45 | PR |
| 9 | III | C/C (5x) | 9.21 | PD |
| 10 | III | C/C (4x) | 3.28 | PD |
| 11 | III | C/C (5x) | 2.61 | PD |
| 12 | III | Cy (1x) | 3.60 | PD |
| 13 | III | CC (6x) | 0.31 | PR |
| 14 | III | Cy (4x) | 2.39 | PD |
| 15 | III | Cy (5x) | 5.36 | SD |
| 16 | III | CC (4x) | 3.15 | CR |
| 17 | III | CC (6x) | 2.14 | PR |
| 18 | III | CP (1x) | 1.85 | PD |
| 19 | III | CC (4x) | 3.35 | CR |
| 20 | III | CC (5x) | 2.68 | PD |

Pts., patients; GST pi, μg mg$^{-1}$ cytosolic protein; CC, cyclophosphamide, carboplatin; CP, cyclophosphamide cisplatin; Cy, cyclophosphamide; CR, complete remission; PR, partial response; SD, stable disease; PD progressive disease.

**Figure 4** The relations between the enzymatic activity towards CDNB of cytosolic glutathione S-transferase and the amount of GST pi in the various tumour samples (■ = untreated cancers, Δ = residual disease, O = recurrent disease).

**Figure 6** Relation between the quantity of glutathione S-transferase (amount of GST pi) and the response to platinum/cyclophosphamide treatment.

group of eight patients with residual disease, from which tumours were obtained after chemotherapy, progressive disease occurred in all patients despite second and sometimes third line chemotherapy (various regimens). In the group of eight patients with recurrent disease after first line chemotherapy progressive disease occurred in five patients during second line chemotherapy (various regimens), one patient was lost for follow-up, and complete remission after second line chemotherapy occurred in two patients (one patient received six courses of methotrexate, and one patient six courses of cisplatin/cyclophosphamide). Recurrence of disease after first line chemotherapy in these last two patients occurred after 4 and 5 years, respectively. In the patients with residual or recurrent disease no relation was found between GST pi levels in the tumour specimens and response to second line chemotherapy.

### Discussion

Response to platinum/cyclophosphamide chemotherapy in ovarian cancer is quite variable. Well known prognostic factors in ovarian cancer are FIGO stage, differentiation grade, age of the patient, histopathologic type, residual tumour load after first laparotomy, morphometrical features and cellular DNA content (Baak *et al.*, 1988). However, even within a group of patients with the same prognostic factors the response to chemotherapy is unpredictable. Therefore additional markers are needed to predict response to chemotherapy. Recently, a positive correlation between the enzymatic activity of GST and drug resistance to platinum and/or alkylating agents has been reported in cell lines (Lewis *et al.*, 1988; Meyer *et al.*, 1990*a*; Teicher *et al.*, 1991; Ali-Osman *et al.*, 1990; Ford *et al.*, 1991). Cell lines with *in vitro* acquired resistance or cell lines derived from resistant tumours showed higher GST levels than their non-resistant equivalents. Furthermore, expression of GST genes in cell lines led to a resistance against these types of compounds (Moscow *et al.*, 1989*a*). These observations suggest a role of GST in acquired drug resistance, possibly by means of an increased glutathione conjugation of antineoplastic agents or their reactive intermediates. Repeated treatment of patients with malignant ovarian tumours with platinum and/or alkylating agents may induce overexpression of GST. In this study both GST enzymatic activity and GST pi (which was the dominant GST isoenzyme) level were lower after treatment with platinum/cyclophosphamide in comparison to untreated tumours. However, as in most *in vivo* studies, also our study is characterised by several complicating factors. Even in the patients with residual disease after chemotherapy the period between last course of chemotherapy and time of excision of the tumour specimen was at least 4 weeks, and therefore a possible transient rise in GST levels may be missed. Our series is small, and the range of histological types of tumours is wide. In only two patients tumour specimens were obtained before and after chemotherapy. GST pi levels were lower after chemotherapy in these two patients with paired specimens, while in one patient GST activity was lower, and in the other GST activity was higher after chemotherapy.

Lower GST activity in malignant ovarian tumours after chemotherapy is in agreement with the findings of Djuric *et al.* who found decreased activity of GST in malignant ovarian tumours after platinum/cyclophosphamide chemotherapy (Djuric *et al.*, 1990). In their study isoenzyme patterns of GSTs, and relations of GST pi level to response to chemotherapy were not determined.

In this study the acidic pi class GST was the most abundant GST form in benign ovarian tumours, and in malignant ovarian tumours, as was found by others in different human tumours, including lung, colon, bladder, and breast tumours (Di Ilio *et al.*, 1985; 1988; Carmichael *et al.*, 1988; Shea *et al.*, 1988; 1990). Lewis *et al.* (1989) described extremely high levels of the alpha class subunit in one malignant ovarian tumour, but this finding could not be confirmed by us in 36 malignant ovarian tumours. The isoenzyme composition in the residual and recurrent malignant ovarian tumours after platinum/cyclophosphamide chemotherapy did not change in comparison to the untreated malignant ovarian tumours. No other data exist in literature regarding GST isoenzyme patterns in malignant human tumours before and after chemotherapy. GST pi levels correlated well with GST activity in treated and untreated ovarian tumours (Figure 4). The one outlying point belongs to the patient with specimens obtained before and after chemotherapy, higher GST activity and lower GST pi level after chemotherapy. The isoenzyme pattern of the residual tumour of this patient showed no marked differences as compared to the corresponding primary tumour. Although one explanation for the disparity in GST activity and GST pi level may be a 'missed' isoenzyme in the HPLC assay, in our opinion this observation cannot be explained by the presence of major amounts of other isoenzymes.

Mean GST pi level was higher ($P < 0.05$) in untreated malignant ovarian tumours compared to benign tumours. This is in agreement with the findings for other human tumours, such as lung, colon, bladder, and breast tumours, where GST pi levels were higher in malignant tissue in comparison to the adjacent benign tissues (Shea *et al.*, 1988; Moscow *et al.*, 1989*b*; Howie *et al.*, 1990). However, mean GST pi level was equal in benign ovarian tumors and malignant ovarian tumours after platinum/cyclophosphamide chemotherapy.

Above mentioned *in vitro* studies suggested a relation in GST pi levels and resistance to chemotherapy. Uncertainty remains however, as to whether data describing mechanisms of resistance *in vitro* are relevant in human tumours. In earlier work we described no changes in P-glycoprotein and lower topoisomerase II in malignant ovarian tumours after platinum/cyclophosphamide chemotherapy in comparison to untreated tumours (van der Zee *et al.*, 1991). In the current study no relation could be found between GST pi levels in untreated malignant ovarian tumours and response of these patients to platinum/cyclophosphamide chemotherapy. So far no further data exist in literature on GST pi levels in tumours and response to platinum/cyclophosphamide chemotherapy. In patients with human breast tumours no relation was found between GST pi expression and *in vitro* chemosensitivity to doxorubicin (Keith *et al.*, 1990), and in another study in human breast tumours no relation was found between GST pi content and other prognostic factors (Shea *et al.*, 1990). Kim *et al.* also did not find GST pi expression as an indicator of response to adriamycin in 15 human tumours (Kim *et al.*, 1991). Lower levels of GST pi after platinum/cyclophosphamide chemotherapy and no relation of GST pi levels with response to platinum/cyclophosphamide chemotherapy both do not suggest an important role of GST pi in *in vivo* drug resistance. However, for the assays, as used in our study, homogenisation of tissue is required. In this way small subpopulations of tumour cells with high GST pi levels between large populations of tumour cells with low GST pi levels can be missed. Terrier *et al.* found heterogeneity of expression of GST pi among different normal human tissues and also heterogeneity of GST pi expression within the same tissue using an immunohistochemical detection technique (Terrier *et al.*, 1990). Recently Rahilly *et al.* also found heterogeneity of GST isoenzyme expression in benign and untreated malignant ovarian tumours (Rahilly *et al.*, 1991). Perhaps the small subpopulations with high GST pi levels will eventually determine the response of the tumour to chemotherapy, and therefore, although not in favour for a significant role for GST pi as marker of clinical drug resistance, out study does not rule out a possible role of GST isoenzymes in clinical drug resistance. In the future determination of GST pi levels with HPLC as well as an indirect immunohistochemical technique, using a polyclonal antibody against GST pi, can elucidate this problem. Another complicating factor in evaluating GST and glutathione levels in tumour specimens is the fact, that GSTs and glutathione are parts of a complicated detoxification system, of which glutathione and GSTs steady states are often used to determine the level of this detoxification system, as was done for GSTs in the current study. Insight in the kinetics of glutathione and GST status should give a more dynamic representation of the continuous availability of this defence (Meijer *et al.*, 1990*b*). However, our findings, that GST pi levels in untreated tumours showed no relation to response to chemotherapy, and that GST pi levels were decreased in malignant tumours after platinum/cyclophosphamide chemotherapy make the eventual use in clinical trials of inhibitors of GST enzymes, such as ethacrynic acid or piriprost as modulation strategy in enhancing sensitivity of malignant tumours to platinum/cyclophosphamide chemotherapy not very promising (Schilder *et al.*, 1990). Our study indicates that other *in vitro* well established mechanisms of drug resistance, such as decreased cell membrane transport of platinum, other detoxification pathways, and changes in repair of platinum DNA adducts, need to be evaluated *in vivo*, as was done for GSTs in the present study.

We greatly acknowledge all participating pathologists and gynaecologists for their help in collecting tumour samples.

This study was supported by grant GUKC 90-18 and TNOV-92-93 of the Dutch Cancer Society.

### References

ALI-OSMAN, F., STEIN, D.E. & RENWICK, A. (1990). Glutathione content and glutathione S-transferase expression in 1,3-bis(2-chloroethyl)-1-nitrosourea-resistant human malignant astrocytoma cell lines. *Cancer Res.*, **50**, 6976.

ANDREWS, P.A. & HOWELL, S.B. (1990). Cellular pharmacology of cisplatin: perspectives on mechanisms of acquired resistance. *Cancer Cells*, **2**, 35.

BAAK, J.P.A., SCHIPPER, N.W., WISEE-BREKELMANS, E.C.M., CEELEN, Th., BOSMAN, F.T., VAN GEUNS, H. & WILS, J. (1988). The prognostic value of morphometric features and cellular DNA content in cis-platin treated late ovarian cancer patients. *Br. J. Cancer*, **57**, 503.

BOYER, T.D. (1989). The glutathione S-transferases: an update. *Hepatology*, **9**, 486.

BOGAARDS, J.J.P., VAN OMMEN, B. & VAN BLADEREN, P.J. (1989). An improved method for the separation and quantification of glutathione S-transferase subunits in rat tissue using high performance liquid chromatography. *J. Chromatogr.*, **474**, 435–440.

CARMICHAEL, J., FORRESTER, L.M., LEWIS, A.D. & 4 others (1988). Glutathione S-transferase isoenzymes and glutathione peroxidase activity in normal and tumour samples from human lung. *Carcinogenesis*, **9**, 1617.

DI ILIO, C., SACCHETTA, P., DEL BOCCHIO, G., LA ROVERE, G. & FREDERICHI, G. (1985). Glutathione peroxidase, glutathione S-transferase and glutathione reductase activity in normal and neoplastic breast tissue. *Cancer Lett.*, **29**, 39.

DI ILIO, C., DEL BOCCHIO, G., ACCETO, A., CASACCIA, R., MUCILLIA, F. & FEDERICI, G. (1988). Elevation of glutathione transferase activity in human lung tumor. *Carcinogenesis*, **9**, 335.

DJURIC, Z., MALVIYA, V.K., DEPPE, G. & 5 others (1990). Detoxifying enzymes in human ovarian tissues: comparison of normal and tumor tissues and effects of chemotherapy. *J. Cancer Res. Clin. Oncol.*, **116**, 379.

FORD, J.M., HAIT, W.N. & MATLIN, S.A. (1991). Modulation of resistance to alkylating agents in cancer cells by gossypol enantiomers. *Cancer Lett.*, **56**, 85.

HABIG, W.H., PABST, M.J. & JAKOBY, W.B. (1974). Glutathione S-transferases. *J. Biol. Chem.*, **249**, 7130.

HOWIE, A.F., FORRESTER, L.M., GLANCEY, M.J. & 5 others (1990). glutathione S-transferase and glutathione peroxidase expression in normal and tumour human tissues. *Carcinogenesis*, **11**, 451.

KEITH, W.N., STALLARD, S. & BROWN, R. (1990). Expression of mdr1 and gst-pi in human breast tumours: comparison to *in vitro* chemosensitivity. *Br. J. Cancer*, **61**, 712.

KIM, R., HIRABAYASHI, N., NISHIYAMA, M., SAEKI, S., TOGE, T. & OKADA, K. (1991). Expression of MDR1, GST-π and topoisomerase II as an indicator of clinical response to adriamycin. *Anticancer Res.*, **11**, 429.

LEWIS, A.D., HAYES, J.D. & WOLF, R.D. (1988). Glutathione and glutathione-dependent enzymes in ovarian adenocarcinoma cell lines derived from a patient before and after the onset of drug resistance: intrinsic differences and cell cycle effects. *Carcinogenesis*, **9**, 1283.

LEWIS, A.D., FORRESTER, L.M., HAYES, J.D. & 5 others (1989). Glutathione S-transferase isoenzymes in human tumours and tumour derived cell lines. *Br. J. Cancer*, **60**, 327.

LEYLAND-JONES, B.R., TOWNSEND, A.J., TU, C.P., COWAN, K.H. & GOLDSMITH, M.E. (1991). Antineoplastic drug sensitivity of human MCR-F breast cancer cells stably transfected with an human alpha class glutathione S-transferase gene. *Cancer Res.*, **51**, 587.

MANNERVIK, B., ALIN, P., GUTHENBERG, C. & 4 others (1985). Identification of three classes of cytosolic glutathione transferase common to several mammalian species: correlation between structural data and enzymatic properties. *Proc. Natl Acad. Sci. USA*, **82**, 7202.

MANNERVIK, B. & DANIELSON, U.H. (1988). Glutathione transferase – structure and catalytic activity. *Critical Rev. Biochem.*, **23**, 283.

MEIJER, C., MULDER, N.H. & DE VRIES, E.G.E. (1990*a*). The role of detoxifying systems in resistance of tumor cells to cisplatin and adriamycin. *Cancer Treat. Rev.*, **17**, 389.

MEIJER, C., MULDER, N.H., HOSPERS, G.A.P., UGES, D.R.A. & DE VRIES, E.G.E. (1990*b*). The role of glutathione in resistance to cisplatin in a human small cell lung cancer cell line. *Br. J. Cancer*, **62**, 72.

MOSCOW, J.A., TOWNSEND, A.J. & COWAN, K.H. (1989*a*). Elevation of pi class glutathione S-transferase activity in human breast cells by transfection of the GST pi gene and its effect on sensitivity to toxins. *Mol. Pharmacol.*, **36**, 22.

MOSCOW, J.A., FAIRCHILD, C.R., MADDEN, M.J. & 7 others (1989*b*). Expression of anionic glutathione S-transferase and P-glycoprotein genes in human tissues and tumors. *Cancer Res.*, **49**, 1422.

OGURA, K., NISHIYAMA, T., OKADA, T. & 5 others (1991). Molecular cloning and amino acid sequencing of rat liver class theta glutathione S-transferases Yrs-Yrs inactivating reactive sulfate esters of carcinogenic arylmethanols. *Biochem. Biophys. Res. Comm.*, **181**, 1294.

OZOLS, R.F. & YOUNG, R.C. (1991). Chemotherapy of ovarian cancer. *Semin. Oncol.*, **18**, 222.

PIVER, M.S., BAKER, T.R., PIEDMONTE, M. & SANDECKI, A.M. (1991). Epidemiology and etiology of ovarian cancer. *Semin. Oncol.*, **18**, 177.

RAHILLY, M., AL NAFUSSI, A. & HARRISON, D.J. (1991). Distribution of glutathione S-transferase isoenzymes in primary epithelial tumours of the ovary. *Int. J. Gynecol. Cancer*, **1**, 268.

SEROV, S.F., SCULLY, R.E. & SOBIN, L.H. (1973). *Histological typing of ovarian tumors*. Geneva: World Health Organization, pp. 17–18.

SCHILDER, R.J., NASH, S., TEW, K.D., PANTING, L., COMIS, R.L. & O'DWYER, P.J. (1990). Phase I trial of thiothepa (TT) in combination with the glutathione transferase (GST) inhibitor ethacrynic acid (EA). *Proc. AACR.*, **33**, 1051.

SHEA, T.C., KELLY, S.L. & HENNER, W.D. (1988). Identification of an anionic form of glutathione transferase present in many human tumours and human tumour cell lines. *Cancer Res.*, **48**, 527.

SHEA, T.C., CLAFLIN, G., COMSTOCK, K.E. & 5 others (1990). Glutathione transferase activity and isoenzyme composition in primary human breast cancers. *Cancer Res.*, **50**, 6848.

SOBRE, B., FRANKENDAL, B. & VERESS, B. (1982). Importance of histological grading in the prognosis of epithelial ovarian carcinomas. *Obstet. Gynecol.*, **59**, 567.

TEICHER, B.A., HOLDEN, S.A., HERMAN, T.S. & 6 others (1991). Characteristics of five human tumor cell lines and sublines resistant to cis-diamminedichloroplatinum (II). *Int. J. Cancer*, **47**, 252.

TERRIER, P., TOWNSEND, A.J., COINDRE, J.M., TRICHE, T.J. & COWAN, K.H. (1990). An histochemical study of pi class glutathione S-transferase expression in normal human tissue. *Am. J. Pathol.*, **137**, 845.

THIGPEN, J.T., BLESSING, J.A., VANCE, R.B. & LAMBUTH, B.W. (1989). Chemotherapy in ovarian carcinoma: present role and future prospects. *Semin. Oncol.*, **16** no. 4, Suppl. 6: 58.

VAN OMMEN, B., BOGAARDS, J.J.P., PETERS, W.H.M., BLAAUBOER, B. & VAN BLADEREN, P.J. (1990). Quantification of human hepatic glutathione S-transferase. *Biochem. J.*, **269**, 609.

VAN DER ZEE, A.G.J., HOLLEMA, H., DE JONG, S. & 5 others (1991). P-glycoprotein expression and DNA topoisomerase I and II activity in benign tumors of the ovary and in malignant tumors of the ovary, before and after platinum/cyclophosphamide chemotherapy. *Cancer Res.*, **51**, 5915.

WAXMAN, D.J. (1990). Glutathione S-transferases: role in alkylating agent resistance and possible target for modulation chemotherapy – a review. *Cancer Res.*, **50**, 6449.